# Linking Health Literacy to Self-Care in Hypertensive Patients with Physical Disabilities: A Path Analysis Using a Multi-Mediation Model

**DOI:** 10.3390/ijerph18073363

**Published:** 2021-03-24

**Authors:** Hye Jin Nam, Ju Young Yoon

**Affiliations:** 1 College of Nursing, Seoul National University, Seoul 03080, Korea; nhyejin@snu.ac.kr; 2 Research Institute of Nursing Science, Seoul National University, Seoul 03080, Korea; 3 Center for Human-Caring Nurse Leaders for the Future by Brain Korea 21 (BK 21) Four Project, College of Nursing, Seoul National University, Seoul 03080, Korea

**Keywords:** health literacy, hypertension, path analysis, physically disabled, self-care

## Abstract

Hypertension has been identified as the most prevalent chronic disease, accounting for the majority of premature deaths in people with physical disability in South Korea. Self-care is vital in controlling high blood pressure. Health literacy has been implicated in self-care behaviors; however, the mechanisms behind this relationship remain unclear. Therefore, the present study aimed to test a hypothetical path model estimating the association between health literacy and hypertension self-care behaviors and to verify the mediating effects of access to healthcare, provider–patient interactions, hypertension knowledge, and hypertension control self-efficacy in hypertensive people with physical disability. In total, 211 hypertensive adults with physical disability completed an online survey. A path analysis using a multi-mediation model was performed using AMOS 17.0 (IBM SPSS Inc., Chicago, IL, USA), and indirect effects were estimated using phantom variables. As a result, the model fit indices were deemed excellent. Significant indirect pathways were determined from health literacy to hypertension self-care behavior via provider–patient interactions, knowledge, and self-efficacy, although no direct association was found between health literacy and self-care behaviors. The study findings supported the importance of provider–patient interactions, knowledge, and self-efficacy, which play a role in linking health literacy and self-care behavior in hypertensive patients with physical disability.

## 1. Introduction

An estimated 1 billion people live with disabilities worldwide, and health disparities for this population have increasingly been recognized recently [1]. They are more likely to experience poor health than people without disabilities, as they tend to engage in un-healthy behaviors, such as smoking cigarettes, and have inadequate physical activity, putting them at risk of chronic diseases [2]. Hypertension is one of the most prevalent chronic diseases in the disability population. Individuals living with disabilities have been determined to have a 2.3 times higher risk of hypertension than those without disabilities [3]. In South Korea, the prevalence of hypertension in people with physical disability was 56.4% in 2017, which was twice as high as that of the general population [4]. Moreover, the mortality rates caused by cerebrovascular disease and hypertension were 7.3 times and 5.0 times higher in the population with disability, respectively [5]. Considering that one of the leading causes of cerebrovascular disease is hypertension, hypertension has been determined to account for a large part of premature death in the population with disabilities.

A well-known key approach to controlling high blood pressure and reducing morbidity and mortality is to engage in hypertension self-care behaviors such as following medical advice, taking medications, and adhering to a healthy lifestyle [6]. Self-care adherence is used to describe the congruence between the recommended practices by healthcare providers and actual behaviors [7]. Self-care therefore involves being aware of one’s self, being able to understand symptoms and problems, and appropriately responding to one’s health demands with the goal of improving one’s condition [8]. In other words, the ability to engage in self-care is compromised when a patient is unable to fully understand their diagnosis and treatment [9]. As such, the patient’s ability to find, understand, and act on health-related information, also known as health literacy, is a determinant of the patient’s self-care behavior [10].

Health literacy is defined as having “the cognitive and social skills, which determine the motivation and ability of individuals to gain access to, understand and use information in ways that promote and maintain good health” [11]. Health literacy comprises the application of a set of skills, including reading, numeracy, comprehending, accessing, and using health-related information in support of health and well-being [10]. It empowers people with skills to improve their health and has important medical and societal implications [12]. Evidence has indicated that health literacy has been associated with the understanding of health issues [9], perceived confidence in disease management [13], subjective health status [14], and hospitalization [8]. It can also affect one’s ability to make health-related decisions, communicate with healthcare providers [15], and use preventive healthcare systems [16]. In the context of hypertension management, studies have indicated that health literacy is a determinant of knowledge of hypertension [7,16] and adherence to hypertension control lifestyles, such as blood pressure monitoring and taking antihypertensive medications [14,17], and is also a predictor of blood pressure control and the development of secondary diseases such as ischemic cardiovascular disease [17].

Individuals with disabilities may have the greatest need for health literacy because they have a complex medical history and needs due to their disabilities. Physically disabled people who have a chronic disease are most likely to seek out health information and frequently interact with their physicians because they must adhere to complex medication and special self-care regimens to avoid complications and improve functioning [18]. Low health literacy may impair their ability to communicate effectively with physicians, understand health-related information, and obtain access to appropriate healthcare, which can lead to inappropriate self-care behaviors [19]. Some studies have highlighted the needs of individuals with intellectual and developmental disabilities in terms of general literacy and communication; however, most research on health literacy has ignored the needs of individuals with physical disabilities [18]. Given the evidence of a role for health literacy and the high prevalence of hypertension in people with physical disability, gaining a greater understanding of the impact of health literacy on self-care is a key priority.

Health literacy involves the multidimensional aspects of mechanisms linking health literacy and self-care behaviors. Paassche-Orlow and Wolf explained the linkage of health literacy to health outcomes at systematic, interactional, and self-care levels in their causal pathway model [20]. These mechanisms are illustrated with three distinctive points, focusing on (a) access and use of healthcare; (b) provider–patient interactions; and (c) patient self-care and how these points in turn influence each other [20]. At each respective point, different factors are involved. Regarding the access and use of healthcare, factors such as acute care orientation, a patient’s navigation skills, self-efficacy, and perceived barriers to care can be influenced by health literacy. At the point of provider–patient interactions, the factors comprise communication skills, knowledge, and participation in decision-making. At the point of self-care, factors such as support technologies, mass media, problem-solving, self-efficacy, and knowledge/skills are suggested, which can affect self-care performance [20].

Paasche-Orlow and Wolf’s model is unique in that it highlights the associations of systemic, interactional, and self-care aspects with health literacy. The model proposed that the link between health literacy and its outcome is due to not only individual attributes such as self-efficacy and knowledge, but also to those attributes of systematic and interactional aspects, including access and use of healthcare and provider–patient interactions [20]. Some researchers have attempted to examine the potential mechanisms through which health literacy impacts disease management based on this model. They reported that disease-specific knowledge and self-efficacy, influenced by health literacy, were the determinants of self-care behavior in patients with heart failure [21,22], diabetes [23], and hypertension [14]. Nonetheless, no prior study extensively included the systematic and interactional aspects of the model, such as the access and use of healthcare and provider–patient interactions. Thus, we sought to validate the applicability of Paasche-Orlow and Wolf’s pathway model in predicting the linkage between health literacy and self-care that goes beyond previous research by including four mediators, i.e., access and use of healthcare; provider–patient interactions; hypertension knowledge; and hypertension control self-efficacy, in hypertensive adults with physical disability.

The aim of this present study was to test a hypothetical path model that estimated the influence of health literacy on hypertension self-care behavior and to verify the mediating effects of access to healthcare, provider–patient interactions, hypertension knowledge, and hypertension control self-efficacy between health literacy and hypertension self-care in hypertensive people with physical disability based on the Paasche-Orlow and Wolf’s pathway model [20]. We hypothesized that health literacy would influence hypertension self-care behaviors. Furthermore, it was hypothesized that health literacy would have an indirect effect on hypertension self-care via access to healthcare, provider–patient interactions, hypertension knowledge, and hypertension control self-efficacy in people with physical disability.

## 2. Materials and Methods

### 2.1. Sampling and Data Collection

From November to December in 2020, a cross-sectional online survey was conducted. Convenience sampling was used to recruit hypertensive patients with physical disabilities. The inclusion criteria were as follows: (a) 18 years of age or older; (b) a person who was diagnosed with a physically disability and thus possessed a Handicapped Welfare Card or certificate of disability registration; (c) a person who was diagnosed with hypertension; and (d) absence of blindness and intellectual disability. According to the criteria of the Korean disability registration system, physical disability is defined as a permanent physical impairment resulting from amputation, joint disorder, limb deformity, spinal cord injury, or a motor disturbance [24].

Prior to commencing the survey, the study was approved by the Institutional Review Board (IRB No. 2010/003-006). The recruitment advertisements were distributed to the community rehabilitation centers in Seoul and were placed on an online community website. This online community consisted of more than 7535 members who mostly had physical disabilities and a common interest in physical disability. Among them, the participants who were eligible to the study criteria autonomously participated in our study and they were asked to self-declare whether they met the eligibility criteria. In compliance with IRB regulations, among the participants who accessed the online survey link, only those who had read and agreed to provide informed consent participated in the survey. The survey took approximately 20 min to complete.

In total, 211 participants completed the survey and were included in the analysis. A sample size above 200 was considered sufficient for path analysis [25].

### 2.2. Measurement

#### 2.2.1. Health Literacy

Health literacy was assessed using the short-version of the Korean health literacy scale (S-KHLS) [26]. The validity of this tool has been evaluated by construct validity and a regression modeling with the Korean health literacy scale [26]. This tool consists of 12 items that assess comprehension, numeracy, and health-related terms. The comprehension and numeracy assessment involves required calculations, such as the next appointment date, medication time, liquid medication dose, and comprehension, such as clinic department and nutrition facts. Additionally, knowledge on health-related terms is assessed using a passage of text about a medical topic with omitted words, and the respondents would select a suitable word to insert in the missing place from 4-multiple choice options. The scores of the S-KHLS range 0–12. The internal consistency score was α = 0.80 in the original paper and α = 0.69 in this study.

#### 2.2.2. Access to Healthcare

Access to healthcare served as our measure of access, and the utilization of healthcare was assessed by the scale of perceived access to care [27]. The Korean version of the perceived access to healthcare scale has been shown to be reliable and valid [28]. The scale used in the study consisted of five items specifically asking about perceived access to care domains including availability, accessibility, accommodation, affordability, and the acceptability of healthcare access. Each item was scored on a 5-point Likert scale. The scores range from 5 to 25, with a higher score indicating increased access to healthcare. Cronbach’s alpha was 0.84 in the original research [27] and 0.56 in this study.

#### 2.2.3. Provider–Patient Interactions

Provider–patient interactions were assessed through a medical decision-making tool which has been shown to be valid [29]. The tool contains seven items that inquire about shared decision-making such as doctor–patient communication, doctor’s expertise, exchange information on health, and the understanding of treatments or medical tests. Each item was evaluated on a 5-point Likert scale, where the scores range from 5 to 35 and high scores indicated a higher degree of involvement in medical decision-making. The tool’s Cronbach alpha was 0.83 in Kim’s study [29] and 0.87 in this study.

#### 2.2.4. Hypertension Knowledge 

Hypertension knowledge was assessed using the revised version of the hypertension knowledge questionnaire [30] developed by Viera, Cohen, Mitchell, and Sloane [31]. The questionnaire was shown to be valid [30]. This 7-item questionnaire assesses the knowledge related to the impact of high blood pressure, prevention, treatment, lifestyle, and blood pressure control. The response options include “yes,” “no,” and “do not know.” The scores range from 0 to 7, and a score greater than 4 can be categorized as a high level of knowledge [30]. In this current study, a continuous scale was used to increase the predictive power of the path analytic model. The questionnaire yielded a reliability coefficient of 0.61 in this sample.

#### 2.2.5. Hypertension Control Self-Efficacy

Hypertension control self-efficacy was measured using the Hypertension Self-Efficacy Scale developed by Park [32]. The validity of this tool has been evaluated [32]. This tool contains 10 items that assess the degree of confidence in healthy lifestyle behaviors to control hypertension. Scores for each item range from 10 for “not confident at all” to 100 for “highly confident”, and the score ranges from 10 to 100. The mean score of the 10 items was used, and a higher score indicated higher self-efficacy. Cronbach’s alpha was 0.72 in Park’s study [32], whereas it was 0.85 in this study.

#### 2.2.6. Hypertension Self-Care

Hypertension self-care behaviors were assessed using the validated 16-item hypertension self-care scale [33]. The scale asks participants to indicate how often they perform certain self-care behaviors such as taking medication, diet control, quitting smoking, limiting alcohol, managing stress, participating in physical activity, and weight control. Each item was scored on a 5-point Likert scale. The total score ranges from 16 to 80, with higher scores indicating higher compliance in terms of hypertension self-care behavior. The reliability coefficient of this self-care instrument was 0.96 in a previous study [33] and 0.80 in this sample.

#### 2.2.7. Demographic and Health-Related Characteristics

Demographic characteristics such as age, gender, marital status, education, monthly household income, and health-related characteristics, such as disability severity, functional limitation, and duration of hypertension, were collected. Disability severity was assessed by asking about their degree of disability according to the Korean disability registration system. Functional limitation was measured using the Washington Group Short-set on Functioning (WG-SS) tool [34]. The tool consists of six items that ask about difficulties that the respondents may have in doing certain activities such as seeing, hearing, walking, remembering or concentrating, washing or dressing, and communicating because of a health problem. Four response options from “no difficulty” to “cannot do at all” were given. The duration of hypertension was assessed by how long ago or the year and the month when they were first diagnosed with hypertension by a doctor or other health professionals. The answers were computed in months by subtracting the time at the first diagnosis from the current time of the survey.

### 2.3. Data Analysis

Data analysis was performed using IBM SPSS Statistics 21.0 (IBM Corp., Armonk, NY, USA) and AMOS 17.0 (IBM SPSS Inc., Chicago, IL, USA). Descriptive statistics including frequencies, percentages, means, and standard deviations were used to describe sample characteristics and summarize study variables. Multiple imputation was used in terms of handling missing values. The normality of the variables was tested by inspecting z-values considering skewness and kurtosis. The acceptable range of z-values for medium-sized samples (50 < *n* < 300) is ±3.29, which indicates a normal distribution [35]. In this study, skewness and kurtosis z-values showed that the variables deviated from the assumption of a normal distribution. To check multi-collinearity among the variables, the correlation coefficient, variance inflation factor, and tolerance were examined. In a path analysis, the most commonly used parameter estimation method is the maximum likelihood (ML); however, ML estimation assumes that the data conform to a normal distribution [36]. Since the data in this study were non-normal, a bootstrap estimation was used as an alternative to evaluate the parameters instead of ML. In the bootstrap approach, a large number of samples are drawn with replacement so that these repeated samples create a sampling distribution based on the central limit theorem [37]. To evaluate the fitness of a hypothetical path model, the *p*-value of the Bollen–Stine (B-S) bootstrap, goodness-of-fit index (GFI), comparative fit index (CFI), normed fit index (NFI), root mean square error of approximation (RMSEA), and Tucker–Lewis indices (TLI) were used. The B-S bootstrap is a method that adjusts probability values for chi-squared tests of non-normal data, and the null hypothesis indicated that the model was correct (*p* > 0.05) [37]. The GFI, CFI, NFI, and TLI exceeding 0.90 and RMSEA below 0.08 indicated a reasonable model fit [38]. Standardized estimates, standard errors, and 95% bias-corrected confidence intervals were also adjusted using bootstrap with 2000 bootstrapping samples.

Phantom variables were used to test the indirect effects among the study variables. In the case of a multi-mediation model, which contains more than two mediators, indirect effects among variables cannot be identified using the bootstrapping method. Instead, the setting of a conversion model using phantom variables allows the indication of specific indirect effects associated with the path of each mediating effect [39]. Phantom variables act as virtual variables, and the variance is fixed to 0. Thus, the conclusion does not alter any coefficient or fitness of the original model [39].

Gender, duration of hypertension, and functional limitation were included as the covariance of self-care behaviors in the path analytic model. Gender and duration of hypertension are well-recognized determinants of hypertension self-care behaviors [40]. The functional limitation measured by WG-SS was used to define the degree of functional limitation in performing daily activities, and it was also a determinant of the ability to practice self-care [19].

## 3. Results

### 3.1. Participant Characteristics

The characteristics of the participants are presented in Table 1. Of the participants, 66.8% were female and 33.2% were male. The mean age was 42.09 ± 8.98 years, ranging from 22 to 76 years. More than half of the participants lived with their spouse (58.3%), while others lived without spouse (41.7%). Most had an education level of high school (51.2%) or above (43.1%). Approximately 18.5% of the participants reported that their monthly household income was less than KRW 1,000,000 (Korean won), while 3.7% of them reported KRW 4,000,000 KRW or more. Thirty-six percent of the participants had a severe physical disability, whereas 64% had a mild physical disability. The participants who had a lot of difficulty or could not do the task at all in at least one of the six core domains in WG-SS were 48.8%. Participants reported having hypertension for an average of 47.03 months (±55.01). The mean score for health literacy, self-care, and access to healthcare were 11.45 ± 0.79, 57.23 ± 7.91, and 16.23 ± 2.87, respectively. The mean scores of provider–patient interactions, knowledge, and self-efficacy were 24.94 ± 4.82, 3.93 ± 1.77, and 66.67 ± 14.42, respectively.

### 3.2. Multi-Collinearity among Research Variables

Multi-collinearity, examined using linear regression, showed that the variance inflation factor ranged from 1.03 to 1.86 and tolerance ranged from 0.54 to 0.99, which indicated no multi-collinearity. Pearson’s correlation coefficients among the main variables including health literacy, access to healthcare, provider–patient interactions, hypertension knowledge, hypertension control self-efficacy, hypertension self-care behaviors, functional limitations, and the duration of hypertension also indicated no multi-collinearity (*r* < 0.8), as shown in Table 2.

### 3.3. Fitness of the Hypothetical Path Model

In the hypothetical path analysis model, an exogenous variable was health literacy, and endogenous variables were access to healthcare, provider–patient interactions, hypertension knowledge, hypertension control self-efficacy, and hypertension self-care. Gender, functional limitation, and the duration of hypertension were included as covariates related to hypertension self-care. Overall, the hypothetical path model demonstrated an excellent model fit, with B-S bootstrap *p* = 0.381, GFI = 0.974, CFI = 0.988, NFI = 0.944, TLI = 0.978, and RMSEA = 0.035.

### 3.4. Effect Analysis of the Multi-Mediation Model

**Figure 1:**
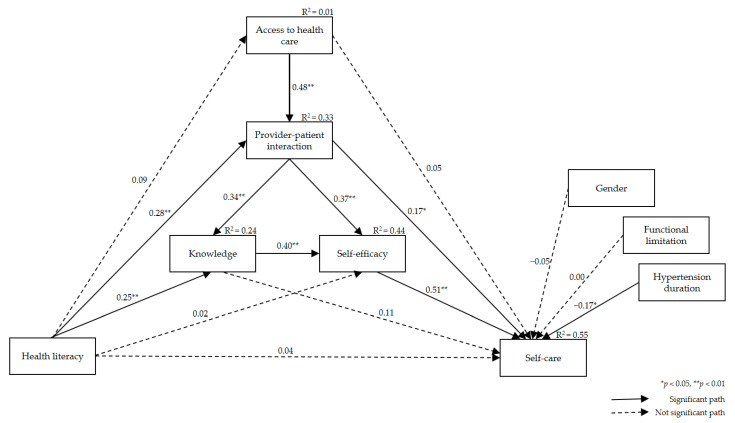
Estimated model with predicted pathways from health literacy to self-care.

Sixteen paths were made in the model to estimate the direct effects among the variables, as shown in Figure 1. Health literacy showed no significant direct effect on hypertension self-care (*β* = 0.04, *p* = 0.383, 95% confidence interval (CI) = −0.05~0.15). Health literacy had significant direct effects on provider–patient interactions (*β* = 0.28, *p* = 0.002, 95% CI = 0.17~0.40) and on hypertension knowledge (*β* = 0.25, *p* = 0.002, 95% CI = 0.08~0.43). Hypertension self-care was found to be directly affected by provider–patient interactions (*β* = 0.17, *p* = 0.021, 95% CI = 0.03~0.29) and hypertension control self-efficacy (*β* = 0.51, *p* = 0.001, 95% CI = 0.38~0.65). Among the pathways within the mediators, access to healthcare had a significant direct effect on provider–patient interactions (*β* = 0.48, *p* = 0.001, 95% CI = 0.39~0.57), while provider–patient interaction had a significant direct effect on hypertension knowledge (*β* = 0.34, *p* = 0.001, 95% CI = 0.20~0.47) and hypertension control self-efficacy (*β* = 0.37, *p* = 0.001, 95% CI = 0.24~0.49). The association between hypertension knowledge and hypertension control self-efficacy was also found to be significant (*β* = 0.40, *p* = 0.001, 95% CI = 0.28~0.51). Regarding covariates, hypertension duration was negatively related to hypertension self-care behaviors (*β* = −0.17, *p* = 0.049, 95% CI = −0.32~0.00). Health literacy explained 33% of the variability in provider–patient interactions, 24% of variability in hypertension knowledge, and 44% of variability in hypertension control self-efficacy. Overall, this model accounted for 55% of the variance of hypertension self-care behaviors.

### 3.5. Indirect Effect Analysis Using Phantom Variables

The phantom modeling, examining the indirect effects of specific paths, identified 12 possible pathways, as shown in Table 3. Among them, four paths were found to have significant indirect effects between health literacy and hypertension self-care. Health literacy had significant, indirect linkage to hypertension self-care through provider–patient interactions (B = 0.48, SE = 0.24, *p* = 0.018, 95% CI = 0.09~1.07). The significant indirect effects emerged in the path from health literacy to hypertension self-care through provider–patient interactions → hypertension knowledge → hypertension control self-efficacy (B = 0.20, SE = 0.07, *p* < 0.001, 95% CI = 0.11~0.39) and through provider–patient interactions → hypertension control self-efficacy (B = 0.54, SE = 0.25, *p* = 0.001, 95% CI = 0.21~1.11). Additionally, the significant indirect effects of health literacy on hypertension self-care were found in the path through hypertension knowledge → hypertension control self-efficacy with a coefficient of 0.53 (SE = 0.29, *p* = 0.001, 95% CI = 0.16~1.24).

## 4. Discussion

The present study has explored the potential pathways linking health literacy to hypertension self-care behavior via theoretically selected mediators (i.e., access to healthcare, provider–patient interactions, hypertension knowledge, and hypertension control self-efficacy) and has further focused on hypertensive patients with physical disabilities. We hypothesized that health literacy would influence hypertension self-care behavior and access to healthcare, provider–patient interactions, hypertension knowledge, and hypertension control self-efficacy would be able to mediate the relationship between health literacy and hypertension self-care behavior. Our path analysis results have partially supported these hypotheses.

First, the participants in this study appeared to have an average hypertension self-care score of 57.2. This was relatively low, compared to those of the hypertensive older adults found in other studies, whose mean scores ranged between 60.0 and 68.1 [32,40]. Self-care behavior has been revealed to be an essential component in the management of hypertension to prevent complications [6]. The low hypertension self-care rate found in this study sample has emphasized that physically disabled people are likely to be at higher risk for multiple complications due to uncontrolled blood pressure; thus, there is an urgent need to develop strategies for improving self-care adherence in people with physical disabilities. The strategies for the inclusion of people with disabilities to fully participate in self-care should focus on understanding barriers to participation and facilitators for building partnerships that support people with disabilities [41]. This study, therefore, attempted to provide a comprehensive understanding of the determinants of self-care behavior, including health literacy, as a potential facilitator or barrier to self-care and relating factors at individual, interactive, and systematic levels. As the proposed variables in our model were able to predict 55% of changes in hypertension self-care behavior; health literacy, provider–patient interactions, hypertension knowledge, and hypertension control self-efficacy should be considered for guiding the planning, development, and delivery of appropriate intervention to improve self-care adherence in this population.

Second, the path analysis has indicated that health literacy was directly related to hypertension knowledge, which, in turn, has been identified as an independent, direct predictor of hypertension control self-efficacy, allowing health literacy to be indirectly related to hypertension self-care. These findings were consistent with prior studies showing that there were significant associations between health literacy and hypertension knowledge [7,14,17] and that self-efficacy was a key determinant of self-care [13,14,22]. Osborn et al.’s study supported the significant paths from health literacy to knowledge, knowledge to self-efficacy, and self-efficacy to self-care behavior in hypertensive patients [14]. Other studies on heart failure patients have also shown independent effects of health literacy on knowledge, self-efficacy and self-care [13,22]. Concerning the relationships between knowledge, self-efficacy, and self-care behavior, individuals must acquire the necessary knowledge and skills so that they may become confident in engaging in particular behavior [14]. Thus, the findings imply that individuals with low health literacy may result in poor self-care behavior, by having deprived knowledge, such as poor comprehension about medication instruction or disease-related information, which can lead to a decreased confidence in disease management.

Although hypertension knowledge and hypertension self-efficacy played a role in mediating the relationship between health literacy and self-care behaviors, non-significant paths from health literacy to self-efficacy and knowledge to self-care were found. Some studies have also found that health literacy predicted self-efficacy [21], whereas others had no association [13,14]. Our results proposed that health literacy may affect hypertension control self-efficacy through hypertension knowledge, but more research is needed to support the valid associations between health literacy and self-efficacy. Likewise, hypertension knowledge was unrelated to hypertension self-care behavior. This finding was congruent with the information–motivation–behavioral skills model explaining that behavioral skills and self-efficacy mainly mediate the effects of knowledge on improving health behavior [42].

Third, provider–patient interactions mediated the indirect relationship between health literacy and hypertension self-care behaviors. This finding was in line with previous studies showing that communication with the provider was related to health literacy and antihypertensive medication adherence [15,43]. Aboumatar et al. identified that hypertensive patients with limited health literacy were reluctant to communicate with their physicians about their medical issues as they had limited ability to understand the medical information [15]. Similarly, other evidence suggested that patients with low health literacy may understand less than half of what is told to them while communicating with their physicians [44]. Patient–physician communication is at the heart of a collaborative relationship between patient and physicians in primary care and is positively related to health behaviors in patients with chronic disease [44]. Although further research is required to discover the specific underlying mechanisms, this study supported a growing body of evidence showing the positive association of provider–patient interactions in relation to health literacy and self-care behaviors.

In the current study, we also confirmed that provider–patient interactions, hypertension knowledge, and hypertension control self-efficacy mediated the relationship between health literacy and hypertension self-care. More specifically, provider–patient interactions predicted hypertension knowledge, and hypertension knowledge directly affected hypertension control self-efficacy. Provider–patient interactions also had a direct effect on hypertension control self-efficacy while mediating the indirect effect of health literacy on hypertension self-care behaviors. Although past studies congruently addressed the associations between knowledge and provider–patient interactions [15] and between self-efficacy and provider–patient interactions [45] resulting in self-care adherence, our results have expanded the previous findings by revealing the mediating effects of provider–patient interactions, knowledge, and self-efficacy between health literacy and self-care using path analysis. Provider–patient interactions are a process in which the provider and patients exchange information, build relationships, and participate in shared decision-making about medical care [15]. The provider’s communication skills are essential for achieving effective provider–patient interactions; however, what is more important is the patient’s health literacy level. This is because the patient’s health literacy can affect their participation and understanding of treatment during interactions with the provider [15]. Patients with low health literacy often hesitate to ask additional questions and rather passively communicate with providers [15]. This tendency is shown because they want to avoid the disclosure of their limited ability to understand medical information. It may eventually result in the patient misunderstanding or misinterpreting health-related information and decreased confidence in engaging in self-care behaviors.

Furthermore, poor provider–patient interactions caused by low health literacy can be more critical for people with disabilities. People with disabilities often have a more complex medical history and issues; thus, they may need additional advice from physicians in order to manage their disease appropriately [18]. Meanwhile, they frequently report finding provider skills as inadequate to meet their needs and being treated badly [19]. Physicians also noted that communication with disabled patients takes more time and is more complex [46]. The interactions between a provider and a patient with disabilities, somehow appeared to be different from the form of interactions with patients without disabilities. Therefore, the characteristics of interactions between a provider and patients with physical disability should be further examined in relation to health literacy status; furthermore, an intervention study is also necessary to develop effective strategies to engage low health literacy patients with physical disability in provider–patient interaction and examine its effects on hypertension knowledge and hypertension control self-efficacy resulting in hypertension self-care.

Fourth, the study findings showed that access to healthcare was not mediating the relationship between health literacy and self-care. This is inconsistent with a previous result suggesting low health literacy was significantly associated with difficulty in finding a provider and access to care needed by community-dwelling adults aged 50 years and older [47]. Unlike the previous finding, the reasons for the insignificant association between access to healthcare and health literacy shown in this study are uncertain, but likely associations can be deduced. Low health literacy is related to an individual’s ability to choose or navigate insurance plans and appropriate healthcare professionals, which can be an access barrier in the general population [16]. In the physically disabled population, people often encounter healthcare accessibility issues because of other types of barriers compared to people without disabilities [48]. The physical or geographical barriers are the foremost access barriers for people with physical disabilities, regardless of their health literacy level [48,49]. For instance, some healthcare institutions possess inadequate facilities, such as lacking railings or slopes, narrow doorways, and inaccessible medical equipment [49]. Additionally, transportation has been identified as one of the factors preventing people with a physical disability from utilizing a healthcare system when needed. For example, they often struggle using public transportation or do not want to be a burden to others in terms of getting to the hospital [50].

Lastly, our findings indicated that health literacy had no direct effect on self-care behaviors though they were indirectly associated. This finding was found to be consistent with a prior study, which reported that health literacy scores were not directly associated with self-care yet were indirectly associated via mediators [14]. In contrast, other research suggested an association between health literacy and hypertension self-care [51]. Although conflicting evidence on the association between health literacy and self-care were found, previous studies congruently demonstrated that self-efficacy and knowledge were significant predictors of self-care adherence [14,16,51]. Adding our results showing indirect effects of health literacy on hypertension self-care behavior via provider–patient interactions, hypertension knowledge, and hypertension control self-efficacy; the mediating factors including provider–patient interactions, knowledge, and self-efficacy may have contributed to untangling the mechanisms underlying health literacy and self-care behavior.

The present study focused on physically disabled people with hypertension and attempted to address pertinent issues related to hypertension management faced by physically disabled people. As a minority group that is vulnerable to inequality in health, research on people with physical disabilities is critically important. Despite the importance of emerging disability studies related to health issues such as chronic diseases, disabled people have largely been excluded from the research field because of low priority and lack of attention [52]. Likewise, their health issues are often dismissed due to the stigma that a disabled person’s health is supposed to be poor [8]. Considering that the number of people with physical disability is growing worldwide because of the aging populations and the rise of chronic diseases, Rio, Magasi, Novak, and Harniss [52] argued that disability should be treated in research as another demographic factor like ethnicity, sex, or age. Our study presented a novel approach in addressing health literacy and hypertension self-care in relation to multiple factors in people with physical disabilities who have hypertension.

This study has several inherent limitations. First, because the study was conducted during the COVID-19 pandemic, the researchers decided to conduct the survey research online to prevent close contact with people according to the government’s safety protocol. This might have restricted the availability of the survey to respondents who were less active online. Although we distributed the survey recruitment advertisements to the community rehabilitation centers as well as to the online community to avoid this limitation, only those who were familiar with using the Internet and had Internet access may have participated in this study. Moreover, it can be anticipated that the participants in this study might have higher health literacy because the literacy level of those who use the Internet with ease may be higher than those who have a limited ability to use the Internet. Therefore, generalizing the study results must be made with caution, and future studies should expand the survey methods and sampling methods such as random sampling or quota sampling to increase generalizability. Second, although the path analysis hypothesized causal relationships between the interested variables based on theory, the cross-sectional nature of this study could limit causal inferences. A prospective study would provide additional insight into the longitudinal effects of these factors on self-care behaviors. Third, some of the study instruments (i.e., access to healthcare and hypertension knowledge) showed low reliability coefficients. The low reliability coefficients might have been due to the number of items on these instruments. For scales with fewer than 10 times, a low Cronbach’s alpha value may be yielded as it is sensitively affected by the number of items in the scale [53]. Another possible reason may be that the instruments did not necessarily perform well in this sample of people with physical disabilities, resulting in lower internal consistency. Thus, future research is required to further validate the instruments in a diverse sample.

Despite these limitations, the findings of this study provided a model for depicting the influence of health literacy on hypertension self-care behavior through access to healthcare, hypertension knowledge, and hypertension control self-efficacy in hypertensive patients with physical disability. This was the first approach that showed comprehensive relationships between health literacy and self-care through multiple mediating factors based on Paasche-Orlow and Wolf’s model. Based on our findings, future studies should extend the findings in diverse contexts of chronic diseases and samples to provide validated explanations for the relationship between health literacy and self-care. In addition, health literacy-focused intervention studies should be conducted to empirically test whether specific provider–patient interactions or knowledge enhances self-efficacy, so that self-efficacy will, in turn, promote the performance of self-care behaviors in hypertensive patients with physical disabilities.

## 5. Conclusions

This study used a multi-mediation path analytic model to identify the effects of health literacy on self-care, mediated by factors at the individual (knowledge and self-efficacy), interactive (provider–patient interactions), and systematic levels (access to healthcare) in hypertensive patients with physical disabilities. Our findings indicated the roles of provider–patient interactions, knowledge, and self-efficacy in linking health literacy with self-care behaviors. While the direct effect of health literacy on hypertension self-care was insignificant, health literacy yielded an indirect effect through provider–patient interactions, hypertension knowledge, and hypertension control self-efficacy on hypertension self-care behaviors. Consequently, the patient’s knowledge of hypertension should be assessed in order to enhance their self-efficacy in performing hypertension self-care behavior when educating physically disabled patients with low health literacy. In addition, the role of provider–patient interactions influenced by health literacy highlighted the idea that improving interactions between providers and patients may be an effective way to increase the patient’s knowledge and self-care behaviors in people with physical disabilities. To sum up, adherence to hypertension self-care behavior in patients with physical disability should be assessed in combination with health literacy, provider–patient interactions, hypertension knowledge, and hypertension control self-efficacy, and intervention programs that consider these factors may be useful for improving hypertension self-care behavior in this group.

## Figures and Tables

**Table 1:** Characteristics of participants (*N* = 211).

Variables	Categories	*n* (%) or Mean ± SD
Gender	Male	70 (33.2)
	Female	141 (66.8)
Age (year)		42.09 ± 8.98
	20~29	11 (5.2)
	30~39	79 (37.4)
	40~49	95 (45.0)
	50~59	14 (6.6)
	≥60	12 (5.7)
Marital status	With spouse	123 (58.3)
	No spouse	88 (41.7)
Level of education	≤Middle school	12 (5.7)
	High school	108 (51.2)
	≥College	91 (43.1)
Monthly household income (KRW 1000)	<100	39 (18.5)
	100~199	62 (29.4)
	200~299	70 (33.2)
	300~399	32 (15.2)
	≥400	8 (3.7)
Disability severity ^1^	Mild	135 (64.0)
	Severe	76 (36.0)
Functional limitation	No	108 (51.2)
	Yes ^2^	103 (48.8)
Duration of hypertension (month)		47.03 ± 55.01
Health literacy		11.45 ± 0.79 (range 6–12)
Hypertension self-care		57.23 ± 7.91 (range 33–74)
Access to healthcare		16.23 ± 2.87 (range 5–25)
Provider–patient interaction		24.94 ± 4.82 (range 9–35)
Hypertension knowledge		3.93 ± 1.77 (range 0–7)
Hypertension control self-efficacy		66.67 ± 14.42 (range 19–100)

^1^ According to the Korean disability registration system. ^2^ Respondents reporting “a lot of difficulty” or “cannot do at all” in at least one of the six core domains such as vision, hearing, mobility, cognition, self-care, and communication; SD = standard deviation.

**Table 2:** Correlations among main variables (*N* = 211).

Variables	X1	X2	X3	X4	X5	X6	X7	X8	X9
	Pearson’s Correlation Coefficient								
Health literacy	1								
Access to healthcare	0.09	1							
Provider–patient interaction	0.32 ***	0.50 ***	1						
Knowledge	0.36 ***	0.20 **	0.43 ***	1					
Self-efficacy	0.28 ***	0.33 ***	0.55 ***	0.57 ***	1				
Self-care	0.28 ***	0.48 ***	0.33 ***	0.53 ***	0.68 ***	1			
Gender	−0.14 *	0.10	0.01	0.08	0.16 *	0.04	1		
Functional limitation	−0.05	−0.05	−0.02	−0.06	−0.05	−0.05	−0.02	1	
Duration of hypertension	0.06	−0.02	0.09	0.13	0.10	−0.09	0.01	0.01	1

X1 = health literacy; X2 = access to healthcare; X3 = provider–patient interaction; X4 = knowledge; X5 = self-efficacy; X6 = self-care; X7 = gender; X8 = functional limitation; X9 = duration of hypertension; * *p* < 0.05, ** *p* < 0.01, *** *p* < 0.001.

**Table 3:** Estimated indirect effects using phantom variables.

Pathways		Estimate	SE	95% CI ^1^		*p*
				Lower	Upper	
Health literacy → Access to healthcare → Self-care		0.05	0.10	−0.07	0.36	0.394
Health literacy → Access to healthcare → Provider–patient interaction	→ Self-care	0.07	0.08	−0.04	0.30	0.206
	→ Knowledge → Self-care	0.02	0.02	−0.01	0.09	0.169
	→ Knowledge → Self-efficacy → Self-care	0.03	0.03	−0.02	0.10	0.229
	→ Self-efficacy → Self-care	0.08	0.09	−0.06	0.29	0.284
Health literacy → Provider–patient interaction	→ Self-care	0.48	0.24	0.09	1.07	0.018
	→ Knowledge → Self-efficacy → Self-care	0.20	0.07	0.11	0.39	<0.001
	→ Knowledge → Self-care	0.11	0.07	0.00	0.27	0.059
	→ Self-efficacy → Self-care	0.54	0.25	0.21	1.11	0.001
Health literacy → Knowledge	→ Self-efficacy → Self-care	0.53	0.29	0.16	1.24	0.001
	→ Self-care	0.28	0.21	−0.01	0.85	0.059
Health literacy → Self-efficacy → Self-care		0.09	0.40	−0.80	0.76	0.818

CI = confidence interval; SE = standard error of indirect effect; ^1^ obtained by bias-corrected percentile method of bootstrapping.

## References

[1] World Health Organization (2020). Disability and Health.

[2] Krahn G.L., Walker D.K., Correa-De-Araujo R. (2015). Person with disabilities as an unrecognized health disparity population. Am. J. Public Health.

[3] Havercamp S.M., Scandlin D., Roth M. (2004). Health disparities among adults with developmental disabilities, adults with other disabilities, and adults not reporting disability in North Carolina. Public Health Rep..

[4] Kim S., Lee Y., Oh W., Hwang J., Oh M., Lee M., Lee N., Oh D., Kang D., Kwon S. (2017). 2017 National Survey of the Disabled Persons.

[5] Ministry of Health and Welfare (2020). 2020 Statistics of People with Disabilities Lives.

[6] Motlagh S.F.Z., Chaman R., Sadeghi E., Eslami A.A. (2016). Self-care behaviors and related factors in hypertensive patients. Iran. Red. Crescent. Med. J..

[7] Son Y., Song E.K. (2012). Impact of health literacy on disease-related knowledge and adherence to self-care in patients with hypertension. J. Korean. Acad. Fundam. Nurs..

[8] Omisakin F.D., Ncama B.P. (2011). Self, self-care and self-management concepts: Implications for self-management education. Edu. Res..

[9] Gazmararian J.A., Williams M.V., Peel J., Baker D.W. (2003). Health literacy and knowledge of chronic disease. Patient Educ. Couns..

[10] Poureslami I., Nimmon L., Rootman I., Fizgerald M.J. (2017). Priorities for action: Recommendations from an international roundtable on health literacy and chronic disease management. Health Promot. Int..

[11] World Health Organization Track 2: Health Literacy and Health Behavior. Proceedings of the 7th Global Conference on Health Promotion.

[12] Berkman N.D., Sheridan S.L., Donahue K.E., Halpern D.J., Crotty K. (2011). Low health literacy and health outcomes: An updated systematic review. Ann. Intern. Med..

[13] Chen A.M., Yehle K.S., Albert N.M., Ferraro K.F., Mason H.L., Murawski M.M., Plake K.S. (2014). Relationships between health literacy and heart failure knowledge, self-efficacy, and self-care adherence. Res. Soc. Adm. Pharm..

[14] Osborn C.Y., Paasche-Orlow M.K., Bailey S.C., Wolf M.S. (2011). The mechanisms linking health literacy to behavior and health status. Am. J. Health Behav..

[15] Aboumatar H.J., Carson K.A., Beach M.C., Roter D.L., Copper L.A. (2013). The impact of health literacy on desire for participation in healthcare, medical visit communication, and patient reported outcomes among patients with hypertension. J. Gen. Intern. Med..

[16] Hall E., Lee S., Clark P.C., Perilla J. (2014). Social ecology of adherence to hypertension treatment in Latino migrant and seasonal farmworkers. J. Transcult. Nurs..

[17] Shi D., Li J., Wang Y., Wang S., Liu K., Shi R., Zhang Q., Chen X. (2017). Association between health literacy and hypertension management in a Chinese community: A retrospective cohort study. Intern. Emerg. Med..

[18] Nguyen J., Gilbert L. (2019). Health literacy among individuals with disabilities: A health information national trends survey analysis. Perm. J..

[19] Johnston M.V., Diab M., Kim S.S., Kirshblum S. (2005). Health literacy, morbidity, and quality of life among individuals with spinal cord injury. J. Spinal. Cord. Med..

[20] Paasche-Orlow M.K., Wolf M.S. (2007). The causal pathways linking health literacy to health outcomes. Am. J. Health Behav..

[21] Dennison C.R., McEntee M.L., Samuel L., Johnson B.J., Rotman S., Kielty A., Russell S.D. (2011). Adequate health literacy is associated with higher heart failure knowledge and self care confidence in hospitalized patients. J. Cardiovasc. Nurs..

[22] Macabasco-O’Connell A., DeWalt D.A., Broucksou K.A., Hawk V., Baker D.W., Schillinger D., Ruo B., Bibbins-Domingo K., Holmes G.M., Erman B. (2011). Relationship between literacy, knowledge, self-care behaviors, and heart failure-related quality of life among patients with heart failure. J. Gen. Intern. Med..

[23] McCleary-Jones V. (2011). Health literacy and its association with diabetes knowledge, self-efficacy and disease self-management among African Americans with diabetes mellitus. ABNF J..

[24] Ministry of Health and Welfare (2013). Policy for People with Disabilities.

[25] Hoe S.L. (2008). Issues and procedures in adopting structural equation modelling technique. J. Quant. Methods.

[26] Lee T.W., Kang S.J. (2013). Development of the short form of the Korean health literacy scale for the elderly. Res. Nurs. Health.

[27] Penchansky R., Thomas J.W. (1981). The Concept of Access. Med. Care.

[28] Gil E., Oh H. (2018). Testing a middle-range theory of self-care of chronic illness: A validation for Korean adult patients with severe hypertension. J. Korean Acad. Nurs..

[29] Kim M.S. (2011). Impact of Shared Decision Making on Patient’s Satisfaction in Rheumatic Disease. Master’s Thesis.

[30] Lim J., Ko G.P., Han E., Jung W., Park M.J., Han J.O. (2012). The Effects Assessment of Chronic Care Management Based on Primary Clinics for Hypertension, Diabetes Patients.

[31] Viera A.J., Cohen L.W., Mitchell C.M., Sloane P.D. (2008). High blood pressure knowledge among primary care patients with known hypertension: A North Carolina Family Medicine Research Network (NC-FM-RN) study. J. Am. Board. Fam. Med..

[32] Park Y. (1994). An Effect of the Self-Regulation, Program for Hypertensives, Synthesis & Testing of Orem and Bandura’s Theory. Ph.D. Thesis.

[33] Lee Y.H. (1995). A study of the effect of an efficacy expectation promoting program on self-efficacy and self-care. J. Korean Acad. Nurs..

[34] Washington Group on Disability Statistics (2020). The Washington Group on Disability Short Set of Disability Questions.

[35] Kim H.Y. (2013). Statistical notes for clinical researchers: Assessing normal distribution (2) using skewness and kurtosis. Restor. Dent. Endod..

[36] Nevitt J., Hancock G.R. (2001). Performance of bootstrapping approaches to model test statistics and parameter standard error estimation in structural equation modeling. Structural Equ. Model..

[37] Bollen K.A., Stine R.A. (1992). Bootstrapping goodness-of-fit measures in structural equation models. Sociol. Methods Res..

[38] Kim G.S. (2013). AMOS Structural Equation Model Analysis.

[39] Macho S., Ledermann T. (2011). Estimating, testing, and comparing specific effects in structural equation models: The phantom model approach. Psychol. Methods.

[40] Kim M., Song M. (2015). Effects of self-management program applying Dongsasub training on self-efficacy, self-esteem, self-management behavior and blood pressure in older adults with hypertension. J. Korean Acad. Nurs..

[41] Ministry of Health (2013). Disability and Primary Health: A Review of the Literacy.

[42] Fisher W.A., Fisher J.D., Harman J. (2003). The information-motivation-behavioral skills model: A general social psychological approach to understanding and promoting health behavior. Soc. Psychol. Found. Health Illn..

[43] Schoenthaler A., Chaplin W.F., Allegrante J.P., Fernandez S., Diaz-Gloster M., Tobin J.N., Ogedegbe G. (2009). Provider communication effects medication adherence in hypertensive African Americans. Patient. Educ. Couns..

[44] Wallace A. (2010). Low health literacy: Overview, assessment, and steps toward providing high-quality diabetes care. Diabetes Spectr..

[45] Lee Y.Y., Lin J.L. (2009). The effects of trust in physician on self-efficacy, adherence and diabetes outcomes. Soc. Sci. Med..

[46] McColl M.A., Forster D., Shortt S.E., Hunter D., Dorland J., Godwin M., Rosser W. (2008). Physician experiences providing primary care to people with disabilities. Health Policy.

[47] Levy H., Janke A. (2016). Health literacy and access to care. J. Health Commun..

[48] Harrington A.L., Hirsch M.A., Hammond F.M., Norton H.J., Bockenek W.L. (2009). Assessment of primary care services and perceived barriers to care in persons with disabilities. Am. J. Phys. Med. Rehabil..

[49] Jeon B., Kwon S., Kim H. (2015). Health care utilization by people with disabilities: A longitudinal analysis of the Korea Welfare Panel Study (KoWePS). Disabil. Health J..

[50] Shin H.I. (2015). Strategic Research for Improving the Disabled People’s Health.

[51] Ahn Y.H., Ham O.K. (2016). Factors associated with medication adherence among Medical-aid beneficiaries with hypertension. West. J. Nurs. Res..

[52] Rios D., Magasi S., Novak C., Harniss M. (2016). Conducting accessible research: Including people with disabilities in public health, epidemiological, and outcomes studies. Am. J. Public Health.

[53] Taber K.S. (2018). The use of Cronbach’s alpha when developing and reporting research instruments in science education. Res. Sci. Educ..

